# Profile of Cytokines TNFα, IL-1β, IL-6, IL-4, and IL-10 in Relation to Disease Progression in a Patient with Advanced Liver Alveolar Echinococcosis and Non-Optimal Antiparasitic Treatment: Four-Year Follow-Up

**DOI:** 10.3390/pathogens14100957

**Published:** 2025-09-23

**Authors:** Katarzyna Zorena, Małgorzata Sulima, Beata Szostakowska, Barbara Siewert, Katarzyna Sikorska

**Affiliations:** 1 Department of Immunobiology and Environmental Microbiology, Faculty of Health Sciences, Medical University of Gdansk, 80-210 Gdańsk, Poland; 2 Division of Tropical Medicine and Parasitic Diseases, Faculty of Health Sciences, Medical University of Gdańsk, 80-210 Gdańsk, Poland; malgorzata.sulima@gumed.edu.pl (M.S.); katarzyna.sikorska@gumed.edu.pl (K.S.); 3 Division of Tropical Parasitology, Faculty of Health Sciences, Medical University of Gdańsk, 80-210 Gdańsk, Poland; beata.szostakowska@gumed.edu.pl; 4 Environment and Health Scientific Circle, Medical University of Gdańsk, 80-210 Gdańsk, Poland; barbara.siewert@gumed.edu.pl

**Keywords:** alveolar echinococcosis, liver, non-optimal antiparasitic treatment, IgE, Em spp. IgG, Em2^+^, TNFα, IL-1, IL-6, IL-4, IL-10

## Abstract

Alveolar echinococcosis (AE) is a zoonotic disease caused by the larval form of the tapeworm *Echinococcus multilocularis*, which is considered one of the most dangerous parasites for humans. *E. multilocularis* infections are most frequently observed in forestry workers, farmers, hunters, berry harvesters, and workers employed in animal shelters. The subject of this study was a four-year follow-up profile of cytokines, including tumor necrosis factor alpha (TNFα), interleukin-1 (IL-1), interleukin-6 (IL-6), interleukin-4 (IL-4), and interleukin-10 (IL-10), in a patient with advanced liver alveolar echinococcosis and non-optimal antiparasitic treatment. Ultrasound, computed tomography (CT) of the abdomen, X-ray, CT of the chest, and magnetic resonance imaging (MRI) of the head were performed during the observation and treatment of the AE patient. After antiparasitic treatment was initiated, decreased activity of the gamma-glutamyl transpeptidase (GGTP), decreased serum concentrations of immunoglobulin E, C-reactive protein (CRP), and the pro-inflammatory cytokines TNFα, IL-1, and IL-6 were observed, as well as slightly increased levels of the anti-inflammatory cytokines (IL-4 and IL-10). Conclusions. During a four-year follow-up in a patient with advanced hepatic alveolar echinococcosis and non-optimal antiparasitic treatment, a decrease in proinflammatory cytokines (TNFα, IL-1β, IL-6) and a slight increase in anti-inflammatory cytokines (IL-4, IL-10) were detected. A better understanding of cytokine regulation in infectious diseases may be important to the development of new therapeutic strategies aimed at antiparasitic treatment. We suggest that broad initiatives (preferably at the local community level) should be implemented to raise awareness of the threat of alveolar echinococcosis and methods for avoiding *E. multilocularis* infection.

## 1. Introduction

Alveolar echinococcosis (AE), caused by the larval form of the tapeworm *Echinococcus multilocularis*, is a rare zoonotic human helminthiasis, but one of the most severe and difficult to treat [1,2]. Adult tapeworms asymptomatically inhabit the small intestine of the definitive hosts, which are wild canids or domestic dogs. Infective eggs produced by adult tapeworms are excreted in the feces of infected animals and contaminate the environment (water and soil). After being ingested by the intermediate host, which in its natural life cycle are numerous species of small, wild rodents, the egg develops into a larva that grows in the abdominal cavity [3,4,5]. A human can become an accidental host via the dirty-hand-to-mouth route (ingestion of eggs in the environment or contact with infected canids) or by eating fruits or vegetables contaminated with parasite eggs [6,7,8]. The World Health Organization (WHO) has listed echinococcosis as one of the 17 neglected diseases targeted for control or elimination by 2050 [9].

The disease is distributed across the Northern hemisphere, where about 17,400 new cases per year are recorded; most of them are in China, but the geographic range is spreading [4,10]. In Europe, most of the described cases come from France, Switzerland, Austria, and Germany, but due to the rising population of the red fox—the main definitive host of *E. multilocularis* in Europe—the parasite, and with it human AE, has been increasingly detected in other European countries, including Poland [8,11,12].

Alveolar echinococcosis may develop asymptomatically over many years. The primary change most often develops in the liver and has a tendency to disseminate to the lungs, mediastinum, brain, and other organs and tissues [12,13]. The literature contains single cases of isolated cerebral alveolar echinococcosis [14], a pineal cyst [15], and ocular echinococcosis [16]. From the moment of infection to the appearance of the first symptoms, several years or more than a decade may pass. A late diagnosis often makes it difficult to save the patient [17]. However, swallowing a tapeworm egg does not always result in disease or death. Moreover, in most cases, the larva calcifies at an early stage of development. It is estimated that only one in ten people exposed to the oncospheres of *E. multilocularis* develops the larva and the disease [2,8,17]. The course of AE is influenced by immunological processes, and previous studies show that by analyzing immunological mechanisms observed in the patient, it is possible to predict whether the treatment is likely to be successful or whether the disease will progress [18].

A healthy human organism strives for a constant balance between T helper 1 (Th1) and T helper 2 (Th2) lymphocyte subpopulations [19,20]. The Th1 lymphocyte subpopulation is associated with protective immunity and the production of interleukin-2 (IL-2), interleukin-12 (IL-12), tumor necrosis factor-alpha (TNF-α), and interferon-gamma (IFN-γ). In turn, the Th2 lymphocyte subpopulation produces interleukin-4 (IL-4), interleukin-5 (IL-5), interleukin-9 (IL-9), interleukin-10 (IL-10), etc., and is associated with susceptibility to infections, pathogenicity, and the survival of parasites in host tissues [18,19]. Both Th1 and Th2 cytokines repair damage induced by pathogens or the immune system, and the expression of Th1 cytokines regulates the balance between fibrosis and injury repair. Macrophages, natural killer (NK) cells, and T lymphocytes—which are stimulated by parasite antigens—produce TNF-α, IL-1, and other inflammatory cytokines [21]. On the other hand, the anti-inflammatory IL-10 and IL-4 are produced by activated macrophages and Th2 cells; they inhibit the production of IFN-γ and increase the production of antibodies, thus shifting the immune response toward Th2. In the response against both intracellular and extracellular parasites, it is IL-4 that plays a key role in the inflammatory response, mediated by immunoglobulin E (IgE) and eosinophils [18,22].

**Figure 1:**
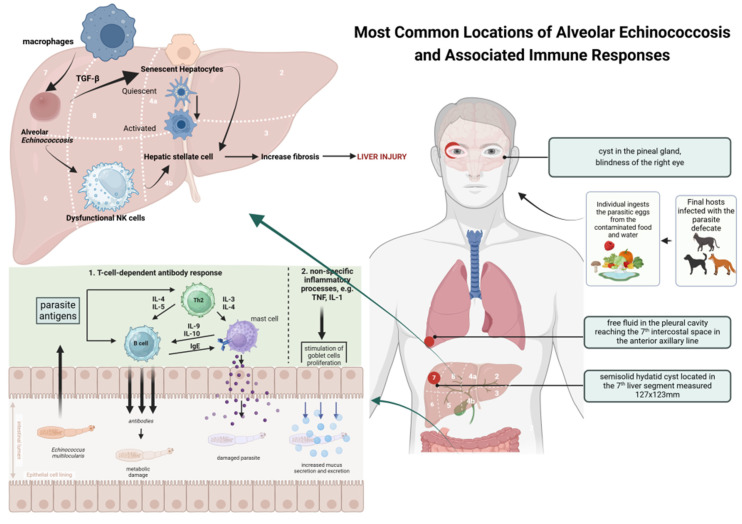
Proposed mechanism of infection and accompanying immune responses in alveolar echinococcosis. Based on [3,7,17,20] and created in BioRender. Siewert B. (2025) https://BioRender.com/uvnadic.

The imbalance between Th1-type cytokines and Th2-type cytokines in AE is not fully understood due to the limited number of studies, regional differences, and complex interactions between parasites and host immunological and genetic factors. Figure 1 shows one of the possible mechanisms in the pathogenesis of alveolar echinococcosis and the accompanying immune responses.

The radical treatment of AE requires partial or extensive surgical resection, combined with long-term use of the anti-helminth drug albendazole [23]. Through its active metabolite, albendazole inhibits cytoplasmic microtubule polymerization and finally diminishes energy production in the cells of adult worms [24]. The relationship between the immune response and albendazole treatment is still poorly understood [25]. The immune system may not only play a general role in the course of parasitic infections but also influence the effectiveness of antiparasitic treatment. With the increasing incidence of AE, a better understanding of the role of Th1 vs. Th2 cytokines during antiparasitic treatment is urgently needed [26,27,28]. Therefore, we attempted to assess the immune profile of Th1 versus Th2, including the involvement of TNFα, IL-1β, IL-6, IL-4, and IL-10 in relation to disease progression in a patient with advanced liver AE.

## 2. Presentation of the Case

**Figure 2:**
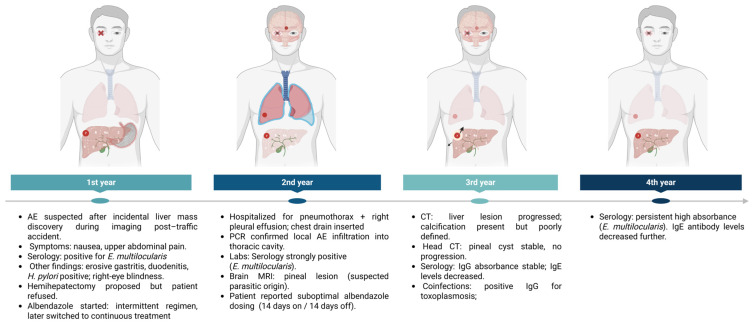
Clinical, immunological, and diagnostic evaluation over four years in liver alveolar echinococcosis under non-optimal antiparasitic therapy. Created in BioRender. Siewert B. (2025) https://BioRender.com/lryhffg.

Temporal evolution of clinical, immunological, and diagnostic findings in a patient with liver alveolar echinococcosis receiving non-optimal antiparasitic therapy. Four-Year Follow-Up (Figure 2).

### 2.1. First Year of Diagnosis and Treatment of a Patient with Liver Alveolar Echinococcosis

A 50-year-old farmer born and still living in a small village in the Warmia-Masuria province in Poland, an endemic area for *E.multilocularis*, was admitted to the hospital (Division of Tropical and Parasitic Diseases, University Centre for Maritime and Tropical Medicine, Gdynia, Poland) with suspected liver AE. The suspected parasitic disease was found accidentally, following abdominal imaging that was performed due to a traffic accident. On initial presentation, the patient complained of periodic, moderately severe nausea and pain in the upper abdomen. The computed tomography (CT) scan revealed an extensive mass in the right lobe of the liver (128 mm × 118 mm). The patient had a positive serological result for *Echinococcus granulosus* IgG. The absorbance for *E.multilocularis* was 2.266, and for *E. granulosus* it was 1.939. Serological test Echinococcus Western blot IgG was used to confirm infection with *E. multilocularis* (Table 1).

Laboratory tests showed no anemia, increased activities of GGTP, ALP, and IgE concentration, and a predominance of gamma fraction in the protein fraction pattern (Table 2). The patient has right-eye blindness, and the underlying cause was most likely a degeneration of the retina and its vasculature (tapetoretinal dystrophy). A chest X-ray did not show any abnormalities. Abdominal ultrasound confirmed a hyperechoic, irregular, and non-homogeneous lesion with a central anechoic zone in the right lobe of the liver. Gastroscopy revealed erosive gastritis and inflammation of the duodenum. There were no signs of recent hemorrhage. A biopsy test detecting *Helicobacter pylori* was positive. The CRP level was elevated, at 13.6 mg/L, and 28 mm/h ESR (Table 2). Moreover, in the blood serum, high levels of TNFα, IL-1β, and IL-6were detected: 2.31 pg/mL, 5.7 pg/mL, and 8.14pg/mL, respectively. However, low levels of IL-4 and IL-10 were detected (1.36 pg/mL vs.0.00 pg/mL), respectively, in a patient with liver alveolar echinococcosis (Table 3).

After surgical consultation, the patient was qualified for a right hemi hepatectomy, though he refused treatment. The patient was started on antiparasitic treatment according to the intermittent treatment regimen that was previously recommended (400 mg of albendazole twice daily for 28 days, followed by a 14-day break). Afterwards, the treatment regimen was changed to continuous in accordance with the recommendations of the World Health Organization Informal Working Group on Echinococcosis (WHO-IWGE) [30].

The patient was discharged home and was recommended to continue antiparasitic treatment and to eradicate the *Helicobacter pylori* infection.

### 2.2. Second Year of Treatment and Observation

In the second year of treatment, the patient was admitted to the Division of Tropical and Parasitic Diseases at the University Centre for Maritime and Tropical Medicine in Gdynia, Poland, following a 4-week hospitalization in the internal medicine and surgical department at his place of residence due to a pneumothorax and fluid in the right pleural cavity. A follow-up chest X-ray showed that these changes persisted. The assay found a pneumothorax with an interpleural distance of up to 45 mm, measured above the level of the fluid; the lung parenchyma had collapsed. Pleural empyema was suspected. A chest CT was performed, a thoracic surgeon was consulted, and a drain was inserted into the right pleural cavity. A polymerase chain reaction (PCR) assay for echinococcosis was positive, indicating local infiltration of AE into the adjacent structures in the chest. Due to the lack of improvement, he was transferred to the thoracic surgery department, where drainage of the right pleural cavity was performed again with the administration of streptokinase to the pleural cavity.

Laboratory tests showed positive ELISA serological test results, including an IgG absorbance for *E.granulosus* of 2.66 and for *E.multilocularis* of 2.573 (Table 1), as well as a high CRP level of 66.33 mg/L and ESR 80 mm/h (Table 2) and serum TNFα, IL-1β, and IL-6 levels of 31.88 pg/mL,0.26 pg/mL, and 7.36 pg/mL, respectively. Serum IL-4 and IL-10 levels were 2.45 and 1.59, respectively (Table 3).

Additionally, in the second year of treatment, the patient underwent head imaging studies (MRI and CT). The MRI of the head showed a focal lesion of possibly parasitic origin in the pineal gland measuring 15 mm × 15 mm × 16 mm; it was hyperintense in T2-weighted images and hypointense in T1-weighted images. Intravenous administration of contrast revealed visible enhancement of the peripheral rim and a capsule covering the mass. There were no signs of other cerebral pathologies, including focal lesions and pathological enhancements. Unfortunately, at this time, the patient declared that he had been taking a lower dosage of albendazole due to financial reasons, changing the dosage schedule to 14 days of taking the drug and a 14-day break.

### 2.3. Third Year of Treatment and Observation

**Figure 3:**
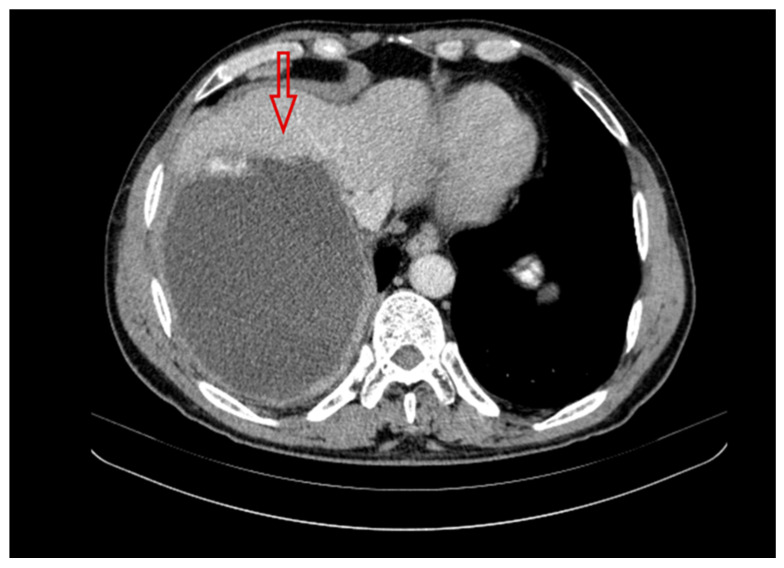
The computed tomography performed in the third year of treatment of a patient with AE. In the right lobe of the liver (red arrow), a parasitic lesion with a dominant fluid component is visible. The contours of the lesion are irregular and contain calcifications. CT photo of a patient treated at the Division of Tropical Medicine and Parasitic Diseases in Gdynia (M.S.; K.S.).

A follow-up CT scan performed in the third year of treatment for the AE patient showed the progression of the parasitic disease; the infiltration in the liver had increased to 142 mm × 138mm (Figure 3). An abdominal CT scan demonstrated the extent of calcification in the abscess, which was poorly defined and became cuboid in shape. There were no other significant changes in the focal lesion from the previous scans. The liver parenchyma was intact.

In the third year of treatment, antibody titers remained at the previous absorbance for *Echinococcus* sp. IgG was 2.362, and for *Echinococcus multilocularis* Em2^+^ it was 2.414. The IgE concentration was lower than in the previous year (Table 1). The patient tested positive for toxoplasmosis (IgG 241.2) and negative for toxocariasis IgG.

Furthermore, lower levels of TNFα, IL-1β, and IL-6 were detected in the patient’s serum (compared to the first year of treatment): 2.05 pg/mL, 2.1 pg/mL, and 4.1 pg/mL, respectively (Table 3). The levels of CRP and ESR were also lower than in previous years (Table 2). Interestingly, a slight increase in the level of Th2 cytokines, i.e., IL-4 and IL-10, was observed: 3.73 mg/l and 1.504 pg/mL, respectively (Table 3).

### 2.4. Fourth Year of Treatment and Observation

In the fourth year of treatment, the patient’s Em spp. IgG and *E.multilocularis* Em2^+^ absorbance remained high (2.252 and 2.588, respectively). However, a lower concentration of IgE antibodies was observed compared to previous years of antiparasitic treatment (Table 1).

Furthermore, the CRP level was 12.5 mg/L, ERS of 20 mm/h, and the TNFα, IL-1β, and IL-6 levels were 1.63 pg/mL, 1.44 pg/mL, and 2.24 pg/mL, respectively. The IL-4 and IL-10 levels were 4.56 pg/mL and 1.52 pg/mL, respectively (Table 2, Table 3).

### 2.5. Patient’s Current Status (2025)

The patient is currently not undergoing follow-up tests. Despite the scheduled follow-up visits to the clinic, the patient did not report. Therefore, the immunological profile, specific antibody levels, and other relevant parameters have not been monitored. In 2025, the patient contacted the authors by telephone and declared that he was being treated by a family doctor (at his place of residence) and that he irregularly takes albendazole at a dosage of 400 mg per day, with breaks of several weeks. He has not performed follow-up imaging tests since the COVID-19 pandemic. The patient lives in a town 280 km away from the clinic, and transport is very difficult for him.

## 3. Materials and Methods

The patient diagnosed with AE underwent laboratory tests to determine the levels of hemoglobin (Hb), red blood cells (RBC), white blood cells (WBC), platelets (PLT), activities of alanine aminotransferase (ALAT), aspartate aminotransferase (ASPAT), gamma-glutamyl transferase (GGTP), and alkaline phosphatase (ALP), as well as the serum concentrations of immunoglobulin E (IgE) and C-reactive protein (CRP), Erythrocyte sedimentation rate (ESR) at the time of hospital admission. In addition, serological tests were performed. An *Echinococcus granulosus* ELISA IgG kit (BordierAffinity Products SA, Crissier, Switzerland) was used to detect the presence of IgG antibodies against larval *Echinococcus* antigens. Then, to confirm the diagnosis and differentiate between cystic echinococcosis (CE) and AE, an *Echinococcus multilocularis* ELISA kit was used to detect the Em2^+^ antigen (Bordier Affinity Products SA, Crissier, Switzerland).

Ultrasound, computed tomography (CT) of the abdomen, X-ray, CT of the chest, and magnetic resonance imaging (MRI) of the head were performed during the observation and treatment of the patient. To clinically assess the patient’s disease progression, we used the scale developed by the World Health Organization’s Informal Working Group of Echinococcosis (WHO-IWGE) [30]. Based on ultrasound imaging, hepatic AE lesions are classified into different PNM types (P = parasitic mass in the liver, N = involvement of neighboring organs, and M = metastasis) [29].

The ELISA kits, used according to the manufacturer’s instructions, are valid when the absorbance of the TBS-Tw sample (no serum blank) is <0.350. After subtracting this blank, the absorbance of the positive control serum should be >1.200 or >1.000, and the absorbance of the negative control serum should be <12% or <10% of the positive control (in the *Echinococcus granulosus* ELISA kit and *Echinococcus multilocularis* ELISA kit, respectively). The absorbance was read at 405 nm on a Jupiter automatic plate reader (Asys Hitech GmBH, Traun, Austria). Both ELISA kits contain a weak positive control (cut-off) to optimally differentiate echinococcosis-positive sera from normal human sera. According to the manufacturer’s interpretation, samples with an absorbance lower than the weak positive control serum are considered serologically negative, and samples with an absorbance higher than the weak positive control serum are considered serologically positive.

Serological test *Echinococcus* Western blot IgG (LDBIO Diagnostics, Lyon, France) was used to confirm infection with *E. multilocularis*

The diagnosis was confirmed molecularly. A 289 bp. fragment of mitochondrial 12S rDNA of *E. multilocularis* was obtained using semi-nested PCR. The cestode-specific primers p60 (forward) and p375 (reverse) and the conditions of the first PCR were selected, as in Myjak et al. [31]. The primer p60 and the *E. multilocularis*-specific primer Em1 (5′ ACA ATA CCA TAT TAC AAC, AAT ATT 3′) were used in the second PCR. Also, the product from the first PCR was diluted 100×. The conditions of the PCR differed from the initial PCR results in the number of cycles (30 instead of 50) and the annealing temperature 52 °C instead of 55 °C).

Serum levels of TNFα, IL-1, IL-6, IL-4, and IL-10 were quantified by enzyme-linked immunosorbent assay (ELISA) using an R&D Systems Quantikine High Sensitivity Human kit (Minneapolis, MN, USA), according to the manufacturer’s protocol. The absorbance of the tested factors was determined by reading at 450 nm on a CHROMATE 4300 automated plate reader (Awareness Technology, Inc., Palm City, FL, USA). The reference curve was generated in accordance with the manufacturer’s recommendations.

## 4. Discussion

This case report presents an assessment of the Th1 vs. Th2 immune profile during the four-year progression of disease in a patient with non-optimal antiparasitic treatment, resulting from a difficult socioeconomic and professional situation. The patient in our study earns his living from agriculture and animal husbandry, which made him susceptible to parasitic infection. During the initial visit, the patient was admitted with suspected AE. Abdominal CT scans revealed a pathological lesion consistent with a parasitic cyst (echinococcosis) in the seventh lobe of the liver. Serological blood tests confirmed the presence of *E. multiocularis*. In addition, the patient was also diagnosed with another parasitic disease, toxoplasmosis, and a bacterial infection, i.e., *Helicobacter pylori*. The patient was started on albendazole at a dosage of 400 mg twice daily for 28 days, followed by a 14-day break. In the second year of treatment, the patient was diagnosed with an empyema of the right lung and molecular testing (PCR) confirmed the presence of *E. multiocularis*.

Furthermore, the patient was diagnosed with neurodegenerative changes in the right eye, which did not reveal signs of parasitic origin on ophthalmological examination. The available literature reports isolated cases of ocular echinococcosis [32,33]. For example, Muftuoglu et al. [33] presented a case of a subretinal hydatid cyst treated with vitreoretinal surgery. Ocular echinococcosis is usually secondary to a systemic infection, and although its incidence is rare, the disease can lead to devastating visual impairment [16,34]. Previous studies have demonstrated that the host’s immune system plays a key role in the response to parasitic infections [14,26]. The body’s immune system may play a general role in the infection and course of alveolar echinococcosis (AE), and it may also influence the effectiveness of drug treatment.

Alveolar echinococcosis infections have been shown to be associated with a chronic inflammatory course with a weak anti-inflammatory response [19,20]. The clinically recognized acute-phase protein is C-reactive protein. The main biological function of CRP is its ability to recognize pathogens and damaged host cells and to mediate their elimination by recruiting complement and phagocytic cells [35]. In the AE patient discussed herein, the CRP level on admission was 13.6 mg/L. In the second year of treatment, when pleural effusion was diagnosed, the CRP level had increased to 64.33 mg/L. In the third and fourth years of treatment, the CRP levels were 9.54 mg/L and 12.5 mg/L, respectively. These CRP values were elevated, as reference values for CRP in healthy individuals range from 0.1 to 3.0 mg/L. The reference values may vary slightly, depending on the laboratory and the testing method used. However, a CRP level below 5 mg/L is considered normal [34,35].

In addition to elevated CRP, the patient demonstrated higher levels of TNFα, IL-1β, and IL-6 in the first year of treatment, when he reported for treatment. However, in the subsequent years, lower levels of the relevant proinflammatory cytokines were detected. These results may indicate that after initiating treatment, there was a decrease in the proinflammatory cytokines, which were engaged in the inflammatory process to eliminate the parasite. Moreover, along with the decrease in IL-1β and IL-6, an increase in IL-4 and IL-10 levels was observed in the patient’s serum. Our results correspond to the results of other researchers [21,36,37]. For example, Weingartner et al. showed increased levels of cytokines (IL-1, IL-6, and TNFα) in liver samples in a mouse model infected with *E. multilocularis* compared to control mice. Albendazole treatment effectively reduced all inflammatory markers to the levels detected in uninfected control mice [37]. In turn, Naik et al. showed that in patients with AE who responded to pharmacological treatment, the levels of the anti-inflammatory cytokines IL-4 and IL-10 significantly increased, whereas in individuals who did not respond to treatment, the levels decreased [21]. The patient presented by our team was treated with albendazole. The antibodies’ absorption to Em spp. IgG and Em2^+^ were maintained throughout all four years of treatment (Table 2). The IgE antibody concentration was 6672 mg/L in the first year, the highest level in the four-year observation period. Although a decrease in IgE antibodies was observed during treatment, absorbance to Em spp. IgG and Em2^+^ were still high. Grüner et al. [18] showed in their studies on mice that antibodies are produced during intestinal helminth infections, mainly of the IgE class. The antibodies coat the surface of the parasite’s body, leading to the stimulation of IgE receptors. The inflammatory mediators released during activation accelerate intestinal motility and stimulate intestinal epithelial cells to overproduce mucus, thereby facilitating the elimination of the parasite from the gastrointestinal tract. Furthermore, eosinophils also secrete mediators that directly damage parasite cells, including eosinophil cationic protein and eosin peroxidase [18]. However, in cases of impaired immune function, in which the immune system is unable to effectively protect the body against pathogens and bacteria, the parasite is not completely removed [22,24] (it should be noted that our patient was also infected with *Toxoplasma gondii* and *Heliobacter pylori*). Additionally, it should be noted that the patient was started on albendazole according to the intermittent treatment regimen used at that time: 400 mg of albendazole twice daily for 28 days with a 14-day break [30]. The nonoptimal treatment of alveolar echinococcosis, as in the case of this patient, most often results from a difficult financial situation and/or a lack of family support, as well as the patient’s understanding of the consequences of taking the drug in partial doses. Albendazole is not paid for by the National Health Fund in Poland, and the treatment, which in advanced (late-diagnosed) cases must last many months or years, often exceeds the patient’s financial capabilities. The most common sufferers of AE in Poland are residents of the poor, rural northeastern areas of the country, which are endemic to *E. multilocularis*. According to the WHO [30], the most optimal treatment for AE patients is radical surgery, combined with a time-limited pharmacological treatment with benzimidazole derivatives, usually lasting 2 years. In the remaining patients, long-term pharmacological treatment is necessary. The drug of choice in AE treatment is albendazole [30].

## 5. Limitations of the Study

In our presentation of the case, the authors point out several limitations. The main limitation lies in its single-patient design, which prevents generalization of the findings and makes the observed immunological trends patient-specific. As a single-patient case report, the interpretation of cytokine dynamics (Th1 vs. Th2) is inherently limited by the absence of reference intervals (while such constraints are typical for this study design), they nevertheless restrict the ability to determine the clinical significance of the observed values. Moreover, the patient’s co-infections with *Toxoplasma gondii,* and *Helicobacter pylori*, as well as complications such as empyema, act as confounders that may independently influence immune responses, making it difficult to attribute changes solely to alveolar echinococcosis progression or treatment. And finally, the patient’s irregular adherence to albendazole therapy introduces variability that complicates assessment of the drug’s immunological effects.

## 6. Conclusions

During a four-year follow-up in a patient with advanced hepatic alveolar echinococcosis and non-optimal antiparasitic treatment, a decrease in proinflammatory cytokines (TNFα, IL-1β, IL-6) and a slight increase in anti-inflammatory cytokines (IL-4, IL-10) were detected. However, the absorbance of Em spp. IgG and Em2^+^ *E. multilocularis* remained high. The lower concentration of IgE antibodies was observed compared to previous years of antiparasitic treatment. Further studies are needed on a larger number of patients, without additional parasitic or bacterial burdens, to fully elucidate the cytokine profile in patients with AE.

## Figures and Tables

**Table 1:** Serological antibody results in a patient with AE during a four-year follow-up.

Parameters	Patient Hospitalization at Four Time Points			
	**1st**	**2nd**	**3rd**	**4th**
**Em spp. IgG**	2.226	2.663	2.362	2.252
**Em2^+^**	1.939	2.573	2.414	2.288
*Echinococcus* WB IgG	Positive profile P3			
**IgE IU/mL**	6672	-	2123	1533

Abbreviations: Em spp. IgG- *E. multilocularis* IgG; WB IgG—Western blot Immunoglobulin IgG; IgE- Immunoglobulin IgE.

**Table 2:** Clinical characteristics of the patient with alveolar echinococcosis.

Parameters	Patient of the Treatment and Observation at Four Time Points				Reference Value
Stage of disease [PNM scale] #	PN0M	PN1M0	PN1M0	PN1M0	-
Pharmacotheray	Albendazol	Albendazol	Albendazol	Albendazol	-
Surgical treatment	-	Drainage of right pleural cavity	-	-	-
Radical surgical treatment	-	-	-	-	-
Transplantation	-	-	-	-	-
BMI	26.5	25.5	27.7	27.7	18.5–24.9
RR mmHg	125/80	120/70	125/75	100/60	<120/80
Hb g/dL	13	11.3	15.1	14.3	13.5–17.5
Hematocrit%	40.3	34.4	43.8	42.5	41–53
PLT K/μL	214	342	197	241	150–400
WBC K/μL	4.66	8.45	4.96	5.26	4.0–10.0
Neutrophils%	56	73	61	58.6	40–70
Lymphocytes%	28	15	26	30	20–45
Monocytes%	13	11	11	9.9	2–10
Eosinophils%	3	1	2	1.3	1–6
CRP mg/L ESR mm/1h	13.6 28	64.33 80	9.54 20	12.5 20	<5 <15
ALAT U/L	13	13	12	12	<40
ASPAT U/L	18	14	19	17	<40
GGTP U/L	84	-	23	27	<60
ALP U/L	155	83	74	83	40–129

# According to reference Kern, 2006 [29]. Abbreviations: PNM—P: Hepatic localisation of the Parasite, N: Extra hepatic involvement of neighboring organs; M: The absence or presence of distant Metastasis, P0N0M0—no extra hepatic involvement, no metastasis; P1N1M0—P1 Peripheral lesions without proximal vascular and/or biliary involvement extrahepatic involvement of pleura, no metastasis; BMI—body mass index, RBC—Red Blood Cells, WBC—white blood cells, PLT—platelet count, CRP—C-reactive protein, ESR—Erythrocyte sedimentation rate; ALAT—alanine aminotransferase, ASPAT—aspartate aminotransferase, GGTP—gamma-glutamyl transferase, ALP—alkaline phosphatase.

**Table 3:** Serum cytokine levels in the patient with AE during a four-year follow-up.

Parameters	Serum Cytokine Levels			
	**1st**	**2nd**	**3rd**	**4th**
**TNFα pg/mL**	2.31	1.88	2.05	1.63
**IL1β pg/mL**	5.7	3.26	2.1	1.44
**IL6 pg/mL**	8.14	7.36	4.1	2.24
**IL4 pg/mL**	1.36	2.45	3.73	4.56
**IL10 pg/mL**	0.00	1.597	1.504	1.52

Abbreviations: TNFα—tumor necrosis factor alpha, IL1β—Interleukin 1beta; IL4—Interleukin 4; IL6—Interleukin 6; IL10—Interleukin 10.

## Data Availability

The raw data supporting the conclusions of this article will be made available by the authors on request.
